# Supplementary material for: Clinical Outcome of Rotational Atherectomy in Calcified Lesions in Korea-ROCK Registry
Source: Medicina (Kaunas). 2021 Jul 7;57(7):694. doi: 10.3390/medicina57070694 (PMC8303478; doi:10.3390/medicina57070694)
Supplement: Supplementary file 1 [file medicina-57-00694-s001.zip › medicina-1247366-supplementary.pdf]

Supplementary Appendix

This appendix has been provided by the authors to give readers additional information about their work. Supplement to: K Lee, JH Jung, M Lee et al. “Clinical Outcome of Rotational atherectomy in Calcified lesions in Korea – ROCK Registry”.

Supplement Table S1. Clinical characteristics according to clinical presentation among patients received PCI successfully.

Supplement Table S2. Lesion and procedural characteristics according to clinical presentation among patients received PCI successfully.

Supplement Table S3. Multivariable Cox Regression Analysis of Independent Predictors of all-cause death.

Supplement Figure S1. Kaplan-Meier curves for 3-year clinical outcomes according to clinical presentation.

Supplement Table S1. Clinical characteristics according to clinical presentation among patients received PCI successfully.

	ACS (n=326)	SA (n=215)	p value
Age (years)	72.4 ± 9.6	70.7 ± 9.8	0.049
Male	188 (57.7)	141 (65.6)	0.07
BMI (kg/m^2)	24.0 ± 3.8	24.6 ± 4.0	0.07
Diabetes melltus	183 (56.1)	120 (55.8)	0.94
Insulin use	41 (12.6)	31 (14.4)	0.54
Hypertension	252 (77.3)	171 (79.5)	0.54
Hyperlipidemia	132 (40.5)	110 (51.2)	0.015
Current smoker	62 (19.0)	41 (19.1)	0.99
Chronic kidney disease	61 (18.7)	39 (18.1)	0.87
Prior MI	40 (12.3)	26 (12.1)	0.95
Prior PCI	77 (23.6)	60 (27.9)	0.26
Prior CABG	15 (4.6)	8 (3.7)	0.62
Peripheral vascular disease	17 (5.2)	23 (10.7)	0.017
History of heart failure	47 (14.4)	33 (15.3)	0.77
History of CVA	56 (17.2)	20 (9.3)	0.01
Atrial fibrillation	31 (9.5)	18 (8.4)	0.65
LV EF, %	51.4 ± 13.7	55.1 ± 12.9	0.002

Values are presented as n (%) or mean ± SD.
Abbreviations: SD, standard deviation; ACS, acute coronary syndrome; SA, stable angina; BMI, body mass index; MI, myocardial infarction; PCI, percutaneous coronary intervention; CABG, coronary artery by-pass grafting; CVA, cerebrovascular attack; LV EF, left ventricle ejection fraction.

Supplement Table S2. Lesion and procedural characteristics according to clinical presentation among patients received PCI successfully.

	ACS (n=326)	SA (n=215)	p value
ACC/AHA classification			
Type B2/C	310 (95.1)	196 (91.2)	0.07
Target vessel			0.27
LAD	229 (70.2)	137 (63.7)	
LCX	26 (8.0)	19 (8.8)	
RCA	71 (21.8)	59 (27.4)	
LM disease	51 (15.6)	29 (13.5)	0.49
Multivessel disease	275 (84.4)	167 (77.7)	0.049
Femoral approach	178 (54.6)	120 (55.8)	0.78
IVUS or OCT	139 (42.6)	114 (53.0)	0.018
Number of stents (target-vessel)	1.64 ± 0.69	1.69 ± 0.66	0.40
Mean stent diameter, mm (target-vessel)	2.96 ± 0.35	3.06 ± 0.43	0.004
Stent length, mm (target-vessel)	49.42 ± 21.65	49.99 ± 20.44	0.76

Values are presented as n (%) or mean ± SD.

Abbreviations: SD, standard deviation; ACS, acute coronary syndrome; SA, stable angina; ACC/AHA, American College of Cardiology/ American Heart Association; LAD, left anterior descending; LCX, left circumflex; RCA, right coronary artery; LM, left main; IVUS, intravascular ultrasound; OCT, optical coherence tomography.

Supplement Table S3. Multivariable cox regression analysis of independent predictors of all-cause death.

	Multivariate*	
Variables	HR (95% CI)	p
Acute coronary syndrome	1.94 (1.002-3.75)	0.049
Age	1.04 (1.004-1.08)	0.03
Chronic kidney disease	2.36 (1.24-4.50)	0.009
History of cerebrovascular attack	1.99 (1.03-3.85)	0.041
Use of intravascular image	0.45 (0.23-0.88)	0.021

*adjusted by age, body mass index, current smoker, diabetes mellitus, hyperlipidemia, chronic kidney disease, history of cerebrovascular attack, history of heart failure, presented as acute coronary syndrome, atrial fibrillation, left main disease, multivessel disease, use of intravascular image, and mean stent diameter.
HR, hazard ratio; CI, confidence interval.

Supplement Figure S1. Kaplan-Meier curves for 3-year clinical outcomes according to clinical presentation.

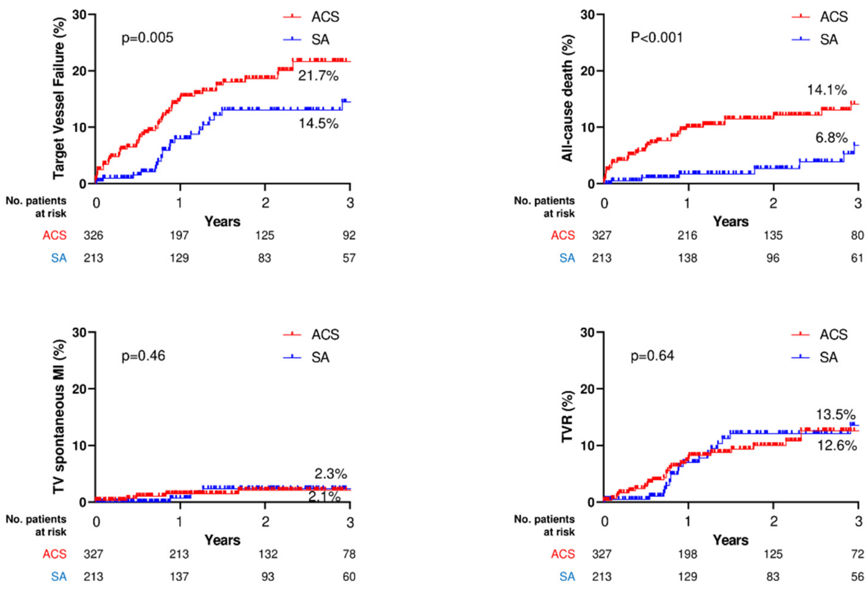

ACS, acute coronary syndrome; SA, stable angina; TV, target-vessel; MI, myocardial infarction; TVR, target vessel revascularization.
